# Seven plant capacities to adapt to abiotic stress

**DOI:** 10.1093/jxb/erad179

**Published:** 2023-05-23

**Authors:** Rana Munns, A Harvey Millar

**Affiliations:** Australian Research Council Centre of Excellence in Plant Energy Biology, School of Molecular Sciences, University of Western Australia, 35 Stirling Highway, Crawley, WA 6009, Australia; Australian Research Council Centre of Excellence in Plant Energy Biology, School of Molecular Sciences, University of Western Australia, 35 Stirling Highway, Crawley, WA 6009, Australia; MPI of Molecular Plant Physiology, Germany

**Keywords:** Cold, deficiency, drought, flooding, heat, nutrient, oxidative, salinity, toxicity

## Abstract

Abiotic stresses such as drought and heat continue to impact crop production in a warming world. This review distinguishes seven inherent capacities that enable plants to respond to abiotic stresses and continue growing, although at a reduced rate, to achieve a productive yield. These are the capacities to selectively take up essential resources, store them and supply them to different plant parts, generate the energy required for cellular functions, conduct repairs to maintain plant tissues, communicate between plant parts, manage existing structural assets in the face of changed circumstances, and shape-shift through development to be efficient in different environments. By illustration, we show how all seven plant capacities are important for reproductive success of major crop species during drought, salinity, temperature extremes, flooding, and nutrient stress. Confusion about the term ‘oxidative stress’ is explained. This allows us to focus on the strategies that enhance plant adaptation by identifying key responses that can be targets for plant breeding.

## Teaser

This review presents seven inherent plant capacities that are essential for reproductive success during the stresses of drought, salinity, flooding, temperature extremes, and nutrient stress.

## Introduction

Climate change and the challenge of feeding an increasing world population pose two existential threats. Climate change causes increased global temperatures, the risk of extreme weather events, rising sea levels, and increased inundation of coastal lands (IPPC, 2022). The increasing world population demands higher productivity of crops and pastures on decreasing areas of traditional agricultural land. Climate change has negatively affected wheat and maize yields for many regions and also the global average. Observed impacts relate mainly to production aspects of food security (IPPC, 2022). Innovative approaches to food production are required.

Abiotic stresses that limit crop yields include drought, salinity, temperature extremes, flooding, and nutrient deficiencies and toxicities. Water is the most limiting resource for crop production, alongside the cost of fertilizer to replace the nutrients extracted by crops. For example, in the USA, soils with low water availability occupy 45% of the agricultural land (Boyer, 1982). Soils that are too wet or too cold for crop production cover another 32%, and only 12% of land is free from physico-chemical problems (Boyer, 1982). Irrigation can supply water for agricultural and horticultural production, but this brings the problem of salinization (Hopmans *et al*., 2021), as well as the competition for water between different usages.

Complex arrays of plant responses to different environmental stresses have been reported over decades. Many reports lack the clear mechanistic basis required to consider them pathways to tolerance, and a basis for plant improvement.

This review focuses on seven capacities possessed by plants that enable them to respond to various abiotic stresses. These are applicable to all plant species, but here we look particularly at their application to food crops.

## Plant capacities essential for abiotic stress responses

The idea of using capacities as a way of thinking about complex and varied biological responses to a changed condition has been used in other scenarios.

The idea of ‘hallmarks of cancer’ as a way of simplifying and applying the information gathered for cancer research was proposed by Hanahan and Weinberg (2000). Rather than add layers of complexity and cancer-specific detail to the literature that is already massive and still accelerating, these authors sought a concept where the complexities of the hundreds of different cancer types could be understood in terms of essential underlying principles. Research had identified a small number of genomic and cellular traits (acquired capabilities) that are shared by most types of human cancer. They suggested six essential alterations in cell physiology that cause malignant growth, including insensitivity to growth-inhibitory signals and evasion of programmed cell death.

More pertinent to plants, Head *et al*. (2015) chose six capacities employed by weeds to invade pristine environments—capacities such as movement, sensing and communicating, flexibility of form, and reproductive timing. These capacities, that enable pioneer and invasive species to compete in new environments with native species, are similar to those we suggest are used by crop species to adapt to abiotic stress. The principles in common with cultivated plants adapting to stresses and the aggressive behaviour of invasive species are flexibility of form and development—that is flexibility of plant architecture and the timing of reproductive growth.

Plant studies often talk about two types of stress—biotic and abiotic (e.g. Dresselhaus *et al*., 2018). These are umbrella terms to separate stress that is caused by a disease (biotic)—where one organism interacts with another and a relationship of dominance is defined—from other stresses that are imposed by abiotic circumstances and environmental factors on a plant. Colloquially, a response of the plant may also be called a stress. The term ‘oxidative stress’ is a case in point. This term is misused, as shown below.

Stress is a physical sciences term referring to a force per unit area; a pressure on materials. The size of the force permits an accurate description and prediction of elastic, plastic, and fluid behaviour of the material. Stress is thus the external force itself, not the response. Strain is the apparent change in the shape, volume, or length of a material caused by stress. A strain can be resisted by a material, can damage or compress a material, or lead to its catastrophic failure (Moebs *et al*., 2016). The effect of strain depends on the capacities of the material in question. In the chemical and biological sciences, the strain relates to changes in the energy status of molecules and the plasticity of form of developing structural components.

Abiotic stresses on plants involve physical and chemical elements that impact their biological functions. Adapted plants show little evidence of strain and thus dominate a niche, or show a perception of stress but adopt strategies to manage consequences, and can complete their life cycle with little loss of seed production (Chase and Leibold, 2003). Non-adapted plants in the same stressful environment show strain by either growing very slowly and barely surviving, or by dying because of a knockout blow (e.g. prolonged heat wave or flood) or the cumulative consequences of debilitating events (Sexton *et al*., 2017).

Conceptually, the extent of plant strain arising from abiotic stress could be understood through the lens of a series of plant capacities that can be recognized in plant function—akin to the properties or capacities of materials under strain in the physical sciences. These could help explain how events affect their life cycle trajectories from germination, establishment, vegetative growth, to flowering and seed maturation. This review seeks to articulate such plant capacities and through them provide a new and simpler framework to consider plant resilience and the strategies for successful plant adaptation. Our vision is to highlight capacities that can provide a framework for sorting and prioritizing the hundreds of genes that are identified in ‘omics’ studies or genome-wide association studies (GWAS). This can accelerate our progress towards increasing food production in regions prone to stressful environments.

The focus on plant capacities that are common to most, if not all, abiotic stresses can reduce the apparent complexity of the response of different species to different environmental conditions, many of which co-exist in nature, for example drought and high temperature stress. This review considers capacities that enable plants to continue growing throughout an abiotic stress, although at a reduced rate.

## The capacities

**Fig. 1.**
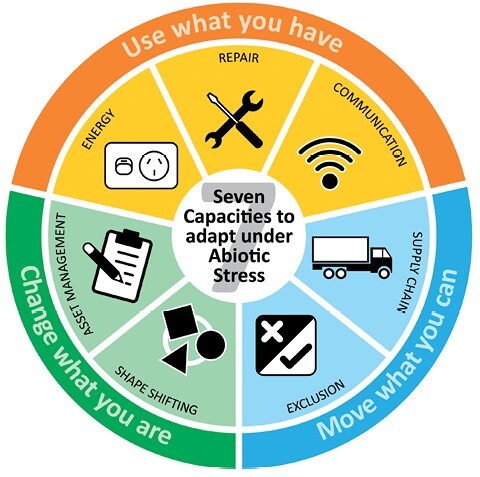
Seven plant capacities that are critical for adaptation to abiotic stress. The inner ring illustrates the seven capacities as icon pictures, the outer ring groups the seven in terms of three approaches to stress tolerance and names each of the seven capacities.

We propose that the outcome of plant responses to abiotic stress can be viewed through the lens of seven capacities and the ability of a plant to enact them collectively to maximize growth and reproductive success. These are the capacities to selectively take up essential resources, store them, and supply them to different plant parts; generate the energy required for cellular functions; conduct repairs to maintain plant tissues; communicate between plant parts, manage structural assets in the face of changed circumstances; and shape-shift through development to be efficient in different environments (Fig. 1).

### Asset-management capacity

The plant’s structural assets are its roots and its leaves, comprised of different organs that grow at different times in development to different sizes and complexities. The structural elements are cells containing solutions that are bounded by a cell wall. Water uptake by cells generates a high internal hydrostatic pressure (‘turgor pressure’) which maintains cell volume and gives strength to the structure as a whole. Management of this structure balances the metabolic need (sources and sinks) and physical need (water and light) for continued growth.

Plants gain capital by using solar energy and carbon dioxide to produce high-energy carbon compounds which can be converted into polysaccharides or proteins, be transported to growing regions of the plant, be stored for later use, or be oxidized to release energy for these purposes.

Best management of this structure results in the optimal balance between transpiration and photosynthesis, between water lost and carbon gained. A shift in the balance between leaf and root growth changes the plant’s evaporative area in relation to the acquisition or retention of water, and the assimilation of nutrients in relation to the carbon budget.

The assets are managed by long-distance signals, namely hormones (Park *et al*., 2017; Beveridge *et al.*, 2023). Local construction is governed by meristem development at the root tips and the shoot apex, and also at sites of lateral root and stem growth. The spatial pattern of cell expansion in the shoot and root growing zones is responsive to water deficit (Yamaguchi and Sharp, 2010) and to salinity stress (Bernstein *et al*., 1993).

The capacity to manage assets occurs throughout the life of a plant as it grows and adapts to adverse environments. For instance, if the soil is deficient in nutrients, the plant can transfer its investment into roots versus leaves, resulting in a change in the root:shoot ratio (Brouwer, 1962). When soil water is limiting further growth, the plant can prolong the life of its current assets, for example delay the senescence of younger leaves by enhancing cytokinin synthesis (Rivero *et al*., 2010). Assets that have become too expensive to maintain such as old roots and leaves can be shed by accelerated senescence. Lastly, the plant architecture can change and be replaced with more suitable structures and models, as shown below.

### Supply chain capacity

Carbohydrates produced by photosynthesis or hydrolysed from reserve compounds are transported by phloem from one tissue to another for growth, respiration, and storage. Without this supply chain, plants cannot connect the outputs and needs of their different organs, or respond to changes in need as development continues or abiotic stress occurs. Tissues that are actively photosynthetic are the ultimate source of these carbohydrates, and it is in these tissues that carbohydrates are loaded into phloem. This loading involves a number of classes of transporters, notably SWEET (Sugar Will Eventually be Exported Transporters) proteins that export sucrose from photosynthesizing cells and SUC/SUT (Sucrose Carrier/Sucrose Transporter) proteins that load sucrose into the phloem (Lemoine *et al*., 2013). Both symplastic and apoplastic routes for sucrose loading to the phloem are found in plants, and their role differs between species and stages of development. Beyond sucrose, other sugar derivatives such as sorbitol, mannitol, and raffinose can also be found and are transported in phloem. Anywhere up to 80% of photosynthetic fixed carbon can be exported by mature leaves to the phloem. The proportion exported depends on triose-phosphates exported from chloroplasts for sucrose synthesis, the rate of storage of sucrose in the vacuole, and the rate of starch synthesis in the chloroplast (Xu *et al*., 2018)

Solute movement in the phloem is governed by the principle of mass flow caused by the pressure difference in the phloem between source and sink tissues. However, along the way, a range of membrane transporters are involved in the cell to cell movement of sucrose and compartmentation between the cytoplasm and vacuoles, providing additional means to regulate sugar fluxes in this supply chain. Sink organs compete for the available sucrose; this so-called sink strength is a combined property of sucrose usage rate and transport process rate. This prioritization system among sinks ensures that roots and young leaves are the major sinks during early developmental stages, whereas tubers and reproductive issues (fruit and seeds) become major sinks later in development. By inducing the conversion of sucrose into starch, SnRK1 facilitates the import of sucrose from the phloem and hence promotes sink strength (Peixoto and Baena-González, 2022). In order for plants to reach maturity and maximize reproductive fitness, priority for access to photoassimilates needs to be established between sinks.

Abiotic stress changes a range of factors that alter this supply chain. Firstly, the sugar synthesis rate can be affected by decreased photosynthetic rates during drought, salinity, or darkness. Secondly, sugars can accumulate in source leaves as a means of osmotic adjustment to maintain metabolic activity but limit supply to sinks (Xu *et al*., 2018). Symplastic loading is considered to be more cold sensitive than apoplastic loading, impacting on the cold sensitivity of symplastic loader species. Supply chain alterations also influence nutrient stress signalling; phosphate limitation signalling in roots is clearly dependent on the sugar transport rate from shoot to root as shown through the discovery that the *pho3* mutant which exhibits constitutive low phosphate signalling is the result of a mutation in the phloem sucrose loader, SUC2 (Lei *et al*., 2011).

### Energy capacity

Cellular processes lead to reductive or phosphorylative costs in the cellular budget that can be measured in REDOX equivalents or ATP requirements. REDOX equivalents are required for many biosynthetic processes that generate a complex chemically reduced product, namely most primary and secondary metabolites. ATP is required for many energy-requiring reactions including nearly all polymer-generating processes in cells, namely complex carbohydrates, lipids, proteins, and nucleic acids. ATP is also needed to maintain membrane potentials across the plasma membrane and many internal membrane systems in cells, and the production of a proton gradient by H^+^-ATPases is needed to pump ions against concentration or charge gradients. While a range of reactions are involved, chloroplasts are the primary generators of cellular NAD(P)H, while both chloroplasts and mitochondria combine as the primary generators of cellular ATP (Raghavendra and Padmasree, 2003; Millar *et al*., 2011).

Both chloroplast and mitochondria function can be lower during abiotic stress, decreasing the generation rate of NAD(P)H and ATP, and limiting cellular energy potential just when energy demands for maintenance functions are increasing (Gururani *et al*., 2015). Having the capacity to generate energy for new demands is important during stress response (Atkin and Macherel, 2009; Millar *et al*., 2011). Alternatively, storage of photosynthates for rapid or delayed release when stress is removed is a valuable strategy for stress tolerance by ensuring energy and reductant are available when required.

It should not be assumed that all transcriptional responses observed during abiotic stress are converted into translational responses. This is evident from varying degrees of correlation between transcript and protein studies in plants (Ponnala *et al*., 2014; Mergner *et al*., 2020) as in other organisms. However, it is also logical from the energy capacity perspective as transcriptional responses are energetically cheap but the translational process is 100 times more expensive (Lynch and Marinov, 2015). Whether the transcriptional response can be implemented in a particular plant tissue depends on the expensive translational and protein homeostasis process that must operate within the energy capacity of cells. One of the reasons that genotype or transcript type does not equal phenotype is this lack of energy availability to enact transcriptional programming, and this is especially an issue during abiotic stress when energy demands are at a maximum.

### Repair capacity

At the molecular level, plant cells are made of complex carbohydrate structures, lipid membranes, proteins, and nucleic acids. Each of these polymers is regularly degraded and replaced to retain the structural, osmotic, and ionic integrity of the cell and the genetic and metabolic capacity to fulfil the physiological fluxes associated with normal plant function (Amthor, 2000). Quality control or repair mechanisms thus retain the steady-state machinery of the cell in optimal condition (Strasser, 2018). For example, in most plant leaves, 10% of each protein pool is replaced daily, while for some proteins this increases to 50% per day or even a complete turnover of a specific protein pool in a few hours (Nelson *et al*., 2014; Li *et al*., 2017). Different tissue types and cell ages also need to repair components at different rates, so plant architecture impacts on the cost and rate of repair rate on a plant basis (Nelson and Millar, 2015).

Abiotic stresses can change the steady-state amount of the polymers that are needed for optimal function and can also increase the degradation rate of components, thus requiring increased rates of repair (Amthor, 2000). These increases in rate for steady states to be maintained can challenge the maintenance capacity of plant cells.

A central component of chemical damage and thus need for molecular repair or replacement is oxidation (Choudhury *et al.*, 2017). Plant cells constantly make a series of reactive oxygen species (ROS) due to catabolic and biosynthetic process that partially reduce O_2_ (Dietz *et al*., 2016). Under ideal conditions, this ROS production is balanced by ROS-scavenging systems to reduce the bulk of ROS before they can oxidize cellular components (Foyer and Noctor, 2009). Cellular components inadvertently and irreversibly oxidized by the remaining ROS are removed and replaced. In the case of some of these cellular reactions, ROS are a minor side product, while in others, partial reduction of O_2_ is the norm and matched ROS scavenging is essential. To accommodate this, separate but interconnected ROS scavenging cycles exist in plastids, mitochondria, peroxisomes, nuclei, and the cytosol. ROS is also produced as the main product of reactions in a range of signalling systems, some of which are involved in plant defence responses to abiotic stress (Mittler, 2002).

During abiotic stress there are a variety of reasons for an increase in ROS production rate, ROS concentration, and ROS-based damage (Mittler, 2002; Foyer and Noctor, 2009). One is the disruption of normal catabolic and biosynthetic processes that partially reduce O_2_ (e.g. changes in temperature differentially altering membrane and soluble reactions). A second reason is decreased availability of NAD(P)H and ATP to maintain or increase ROS-scavenging pathways. For example, drought or flooding lowers the primary reducing equivalent and ATP synthesis pathways in chloroplasts and mitochondria, and extra demands on these fluxes increase, such as energy and reductant requiring biosynthetic reactions to make secondary metabolites like polyphenols, anthocyanins, polyamines, and flavonoids (Ramakrishna and Ravishankar, 2011). A third reason is increases in ROS-based signalling processes (e.g. plasma membrane NADPH oxidase, cellular and extracellular peroxidases). Increased production of ROS will increase the turnover rate of lipid membranes due to free radical reactions damaging acyl chains which will cause membrane leakage if not replaced, while oxidative damage of nucleic acids can cause mutations on replication. Proteins can be damaged by similar oxidative modifications, but increased rates of their catalytic function can also lead to active site damage requiring their replacement (Hanson *et al*., 2021). Protein components can also be damaged by large changes in temperatures, leading to unfolding and proteolysis. The low level of oxygen in flooded soils can dramatically lower the potential for oxidative damage. However, the oxidation risk heightens when flooding recedes and there is a sudden return of oxygen to a plant system that has not maintained an adequate antioxidant defence system, due to limited energy reserves, during a hypoxic or anoxic period.

A high ROS production rate or even a high ROS level is not a sign of damage or of strain in plants *per se*. Actively growing tissues have high levels of both and they can be a normal sign of metabolic activity. Accumulation of unrepaired damage (e.g. accumulation of precipitated proteins, oxidized proteins, peroxidized lipids, or oxidized DNA) is evidence of strain under stress. A more oxidized redox poise of the glutathione and ascorbate pools is early evidence of an overwhelmed antioxidant defence system which if left unchecked can lead to accumulated unrepaired damage. Large antioxidant defence enzyme capacities and large glutathione and ascorbate stores in cells can help prevent over-run or inadequacy following imposition of a stress.

### Communication capacity

Effective communication between plant organs and between different cell types continues throughout the life of the plant and can be modified in response to abiotic stress. Adapting to abiotic stress while maintaining growth and development requires a finely tuned regulatory network that operates at both the whole-plant and cellular level (Zhang *et al*., 2020). Long-distance signals regulate the balance between shoot and root growth, and intracellular signals, such as within the stomatal complex, control gas exchange (Tardieu *et al*., 2018). The ‘stress hormone’ abscisic acid (ABA) is the key regulator of stomatal closure.

Signals can be rapid and short term (hydraulic or electrical) or long term (hormonal), or involve stress signalling networks and cascades between and within cells (as in the stomatal complex). A wide range of communication molecules have been proposed in plants. The most well studied during abiotic stress is the long-distance communication between plant organs by gibberellins (GAs), cytokinins, auxins, strigolactones, ABA, and salicyclic acid (Park *et al*., 2017). Strigolactones interact with auxin to control shoot branching (Beveridge *et al*., 2023). These hormones are mobile in the xylem or phloem or travel via cell to cell pathways.

Evidence also exists for long-distance signalling by ROS cascades (Mittler *et al*., 2011), Ca^2+^ cascades (Dong *et al*., 2022), and sugars in the phloem as mobile signals (Hammond and White, 2008). Intracellular signals then propagate long-distance signals into ROS, SARK (senescence-associated receptor protein kinase), TOR (target of rapamycin), and other signals (Crawford *et al*., 2018, Fu *et al*., 2020) to effect transcriptional events in the cell nucleus that alter cellular function. Within cells, signalling pathways involving membrane transporters, protein kinases, ROS, and Ca^2+^ connect chloroplasts and mitochondria, and provide feedback signals to the nucleus (Kmiecik *et al*., 2016, Crawford *et al*., 2018). These also coordinate the response of plant growth and development to environmental stimuli.

### Developmental shift capacity

The plant’s structural assets, the shoots and roots, capture light and CO_2_, and take up water from the soil. The efficiency of their functions depends on the internal structure of the organs, on the cellular anatomy, and system architecture which alters the shape and form of the organs, and which can be modified throughout the life of the plant. These modifications determine the functional effectiveness of the plant structure.

#### Shape-shifting

**Fig. 2.**
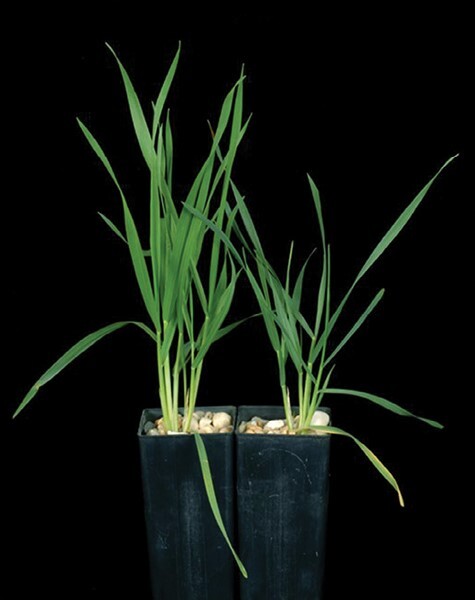
Effect of salinity on wheat seedlings. Wheat plants were grown in supported hydroponics without (left) or with (right) 150 mM NaCl for 10 d. Salinity, like drought, reduces the number of shoots (tillers) and the area of individual leaves. The leaves are thicker and appear darker green as the leaf weight per unit area is greater. Salt toxicity shows in the oldest leaves first. Photo courtesy of Carl Davies.

Shoot architecture can change during the vegetative growth stage to maximize interception of light while minimizing loss of water from leaves; root architecture can change to maximize nutrient and water acquisition. Plasticity of development is important to suit an environment that changes throughout the life cycle of the plant. For example, as the soil dries or becomes more saline, fewer branches or shoots are produced, which is the major restriction on the plant’s evaporative surfaces (Nicolas *et al*., 1993). Further, cell dimensions in new leaves change, leading to smaller but thicker leaves, with greater mass per unit area. Chloroplast density per unit area is therefore greater, and leaves appear a darker green than in unstressed plants (Fig. 2). This may compensate for stomatal closures, and so photosynthesis per unit leaf area may be unchanged. These anatomical changes promote water use efficiency and minimize photoinhibition. Root architecture changes, in that lateral root initiation and elongation are reduced more than seminal root growth resulting in a deeper root system that can access more water at depth (reviewed by Shelden and Munns, 2023). Changes in root anatomy (Zou *et al*., 2021) and the chemical composition of endodermal cell walls (Byrt *et al*., 2018) can modify water and solute transport across roots (Arsova *et al*., 2020). New tools available for phenotyping roots in both controlled and field environments can show adaptive root system architecture for crops facing abiotic stress (Tracy *et al*., 2020).

#### Phase-shifting

Plants build leaf structure and accumulate carbohydrate storage pools during vegetative growth which can continue to be deployed in a shift to reproductive growth. A common response to abiotic stress is an acceleration of the developmental shift from vegetative to reproductive growth. This means that the next primordium formed on the shoot apex does not develop into a leaf but into a floral bud. The timing of reproductive development with respect to the season determines the best ‘use’ of assets for maximum yield and seed quality. This has the result that a seed matures during more benign conditions at the expense of a greater number of late-maturing seeds. This switch may be delayed or even deleted in a perennial crop during adverse environmental conditions.

The phase of flowering, particularly when the anthers emerge, is the most sensitive stage during drought, heat, cold, and salinity stress, leading to the general conclusion that pollen meiosis is the most sensitive process. This has been studied intensively with wheat (Saini *et al*., 1984) and rice (Sheoran and Saini, 1996). The signalling of this phase shift is not fully understood, but the ability to maintain sink strength and carbohydrate supply to anthers is the key to maintaining pollen fertility and grain number in wheat. This phase change may also provide protection against many abiotic stresses (Ji *et al*., 2010).

### Selective uptake capacity

Plant roots can exclude potentially toxic ions in the soil while taking up water and essential ions, protecting plant functions from particular aspects of the chemical environment in which they live. Cells in the epidermis and cortex enhance the uptake of essential nutrients, while limiting the entry of toxic ions. This is done by plasma membrane transporters that are selective for specific ions. Ions do not cross the lipid bilayer of the membrane but enter cells through ion channels (passive transport, down an electrochemical gradient, controlled by opening or closing of ‘gates’) or transporters (active transport, energy provided by proton co-transport).

The electrical potential across the plasma membrane is negatively charged (inside is negative with respect to outside), so energy is needed to take up anions such as phosphate, nitrate, and chloride, as well as potassium if the soil concentration is very low. Several K^+^-specific ion channels and transporters are selective for K^+^ over Na^+^. However, in a saline soil, this selectivity is inadequate to prevent Na^+^ from entering through K^+^ channels and is effluxed by a proton antiporter such as SOS1 (Ji *et al*., 2013). The activity of ion channels is regulated by many cytoplasmic factors that have immediate effects, such as pH and Ca^2+^, and others that modify the channel protein such as ligands, kinases, phosphatases, or calmodulin (Pantoja, 2021).

Selective uptake of soil solutes by roots is the major control point for nutrient entry into the plant, and the net exclusion of potentially toxic compounds. Selective transport of solutes to leaves can take place at three other points— the transport of Na^+^ to the leaves is restricted by the controlled loading of the xylem, withdrawal from the xylem in the upper parts of the roots, and loading of the phloem in leaves (Munns and Tester, 2008).

Ion concentrations of the cytoplasmic compartments are critically important to regulate, especially that of the cytosol as the ion concentrations there determine the fluxes into the other compartments: nucleus, mitochondria, and chloroplast. Potentially toxic ions and organic compounds must be excluded from the cytoplasmic compartments and sequestered in the vacuole. The proton antiporter NHX1 transports Na^+^ and K^+^ into the vacuole (Bassil *et al*., 2019) and is energized by the vacuolar H^+^-pyrophosphatase VP1 (Schilling *et al*., 2017).

The uptake of soil solutes that are extremely toxic is restricted by roots. These include micronutrients that are needed at micromolar concentrations but are toxic at millimolar concentrations such as Zn, Mn, and Fe. These are largely excluded by roots or stored in vacuoles in specific cell types by specific ion transporters (Zhao *et al*., 2022). Soil contaminants such as As and Cd are accumulated by plants but are toxic to animals, so should be excluded by plants used for animal fodder or human consumption.

## The stresses

**Fig. 3.**
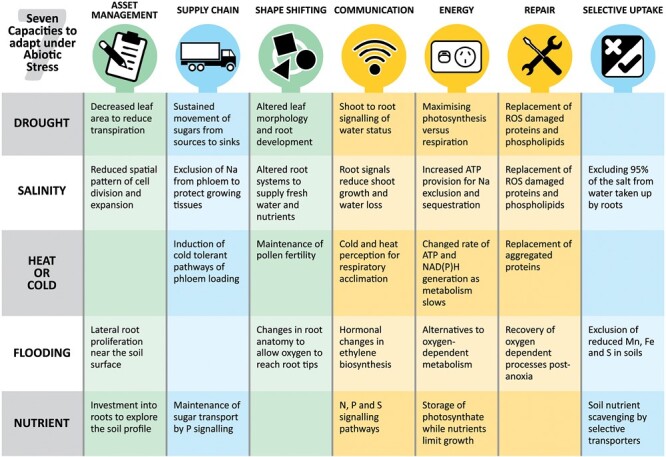
Capacities that are critically important in the response to abiotic stress, and some traits enabled by these capacities. Each environmental stress category has a number of traits that enable a plant to continue growth and produce seed.

To show how these capacities are each relevant to stress responses, we have considered five major types of abiotic stress: drought, salinity, temperature extremes, flooding, and soil nutrient extremes. Drought, salinity and/or high temperature often occur together, while flooding, low temperature, and nutrient deprivation all have unique aspects of how they develop and interact with plant capacities. In the section below, we indicate the capacities that are essential in the adaptation to particular stresses, and refer to examples of traits or genes being used successfully in the field. Some of these traits are highlighted in Fig. 3.

### Drought stress

Drought affects a large spectrum of plant functions such as transpiration, photosynthesis, leaf growth, and reproductive development (Chaves *et al.*, 2003). It impacts the underlying physiological processes including cell division, cell wall mechanics, primary and secondary metabolism, and the repair of oxidized macromolecules. Plants sense the availability of water in the soil, by a mechanism still to be discovered, and send signals to the shoots to reduce both stomatal conductance and leaf expansion so that enough water remains in the soil until plant maturity (Tardieu *et al.*, 2018). Both these reductions limit the photosynthetic ability and carbon supply to the growing tissues. Stomatal closure is an important response to rapid environmental changes, but it causes increases in leaf temperature. In an adapted plant, the growth reduction stays in balance with reduced assimilate production as evidenced by the level of carbohydrate storage pools remaining the same and sometimes higher than in well-watered plants. Osmotic adjustment occurs in all cells, and the cells stay turgid. Long-distance signals between roots and shoots regulate the balance between shoot and root growth, and intracellular signals within the stomatal complex control gas exchange (Skirycz and Inzé, 2010; Tardieu *et al*., 2018). Figure 3 indicates the capacities that are crucial for adapting to the stress.

The water supply must be ‘spared’ or ‘metered’ by the plant if irrigation is not possible, so that adequate water is available during late floral development and grain filling. Such operations often involve trade-offs and risks that must be managed (Passioura and Angus, 2010). The capacities for asset management and developmental shape-shifting are therefore essential components in adapting to drought.

For crops growing in a low-rainfall environment, or over a prolonged period without rain, the most important factor is the efficiency with which the available water is used (Passioura and Angus, 2010). The efficiency of water used to sustain photosynthesis is the fraction of the water supply that is transpired, the efficiency with which the crop exchanges water for CO_2_ in producing biomass (transpiration efficiency; TE), and the fraction of the biomass that ends up in the grain (harvest index). The trait for maintaining carbon supply while limiting evaporative loss by partial stomatal closure (TE) was assessed by carbon isotope discrimination and associated with yield in dry soils (Rebetzke *et al.*, 2002). Three wheat cultivars with enhanced TE were released. All were enriched in TE, but molecular markers were not developed as the trait was multigenic and no genetic effect was large enough.

Many commercial drought-tolerant genetically modified (GM) products relate to the capacities described in the previous section. DroughtGard® is based on a bacterial cold shock protein that contains an RNA chaperone-binding sequence (Castiglioni *et al*., 2008). Other drought tolerance traits for corn include trehalose metabolism (Nuccio *et al*., 2015) and ethylene signalling (Habben *et al*., 2014). Examples of other drought-responsive candidate genes that have been pursued commercially include transcriptional regulators such as dehydration-responsive element-binding (DREB) protein, the feast/famine signalling kinase (SnRK1), and ABA receptors (summarized in Nuccio *et al*., 2018). The likely reason for the failure of the DREB protein to increase yields under drought without a yield penalty in well-watered conditions was the use of the cauliflower mosaic virus (CaMV) 35S promoter which caused pleiotropic growth defects (Nuccio *et al*., 2018).

Application of the capacity for increased supply chain and energy production is illustrated by overexpression of the *RCA* (Rubisco activase) gene which improved fibre yield in cotton under drought and other stresses, with and without co-expression of the Arabidopsis vacuolar pyrophosphatase AVP1 (Pasapulu *et al*., 2011; Smith *et al*., 2023). Drought-induced leaf senescence was delayed in cotton by transformation with the isopentenyltransferase gene (*IPT*) that encodes a rate-limiting enzyme in cytokinin biosynthesis, under the control of a water deficit-responsive and maturation-specific promoter *P*_*SARK*_ (Zhu *et al*., 2018). This improved drought tolerance in cotton (Kuppu *et al*., 2013; Zhu *et al*., 2018).

### Salinity stress

For plants to continue growing in a saline field to maturity, the soil water must be conserved otherwise the salt concentration in the soil solution would continue to increase. As in drought, plants reduce their evaporative area to conserve soil water. Asset management ensures that root growth is favoured over shoot growth, and the root:shoot ratio increases (e.g. Nuttall *et al*., 2006). Fewer branches or shoots are formed (Fig. 2). In addition, the anatomical development of roots is modified (Zou *et al*., 2021), and root system architecture changes (Shelden and Munns, 2023).

Soil salinity imposes two stresses on a plant, a water-deficit stress and an ionic stress (Munns and Tester, 2008). The increase in the Na^+^ and Cl^−^ concentration in the soil solution demands more energy for the plant to take up water while at the same time excluding most of the salt. Roots exclude 95% of the salt in the soil solution while taking up water. In a saline soil where the concentration of Na^+^ may be 100 times greater than that of K^+^, Na^+^ enters via non-selective or K^+^-specific channels, and must be effluxed by a transporter such as SOS1. This ‘net exclusion’ can consume a significant amount of energy (Munns *et al*., 2020). Na^+^ transport to the shoot can be controlled at the point of loading of the xylem by parenchyma cells, and Na^+^ can be withdrawn from the xylem in the upper part of the roots or lower part of the shoot by HKT Group 1 transporters (Schroeder *et al*., 2013) so the amount of Na^+^ reaching the leaves is reduced.

Within cells, Na^+^ is excluded from the cytosol and mitochondria where concentrations should be kept at ~10 mM (Shabala and Munns, 2017). The chloroplast can accommodate higher concentrations of ~100 mM (Bose *et al*., 2017). Na^+^ and Cl^−^ can be stored at high concentrations in vacuoles, especially in halophytes, and supply much of the osmotic pressure needed by cells to balance the osmotic pressure of the salt in the soil. Organic osmolytes balance the osmotic pressure in the different compartments of the cytoplasm (Munns and Tester, 2008). The storage of high concentrations of Na^+^ in vacuoles while keeping very low concentrations in the cytosol requires a considerable amount of energy which is supplied by the vacuolar pyrophosphatase VP1. Transformation of barley with *AVP1* improved the shoot biomass and increased grain yield in a saline field (Schilling *et al*., 2014).

Salinity tolerance has been an active topic of plant science research for many decades, and numerous advances have been made in our understanding of the physiology and molecular genetics of salinity tolerance. However, it is notable that there have been relatively few applications of these research advances to improve performance of crops in salt-affected fields (Melino and Tester, 2023). Examples of the capacity for Na^+^ exclusion are two transporter genes that have been used successfully by breeders to increase salinity tolerance of crops. Quantitative trait loci (QTLs) for low leaf Na^+^ accumulation were found in a rice landrace (*SKC1*, *Saltol*) and a wheat ancestor (*Nax2*). Both genes were identified as Na^+^ transporters of the HKT1 type that remove Na^+^ from the xylem as it flows towards the leaves. They were introgressed by marker-assisted selection into current cultivars of rice and durum wheat, respectively, and shown to increase yield on saline soils in farmers’ fields (Munns *et al.*, 2012; Ismail and Horie, 2017).

### Temperature stress

Temperature stress is a major environmental factor that affects plant growth and development, and influences crop productivity (Wahid *et al.*, 2007). It is now certain that global temperatures will continue to rise, with an increased number of ‘heat events’ (Hunt *et al.*, 2018). The impact of heat waves is worsened by drought. Major heat damage in wheat crops is common globally, causing significant yield reductions (Hunt *et al.*, 2018). Cold temperatures can reduce the yield of ‘tropical’ crops such as rice when grown outside their native environment. Rice suffers from cold injury at the reproductive stage if night temperatures fall below 15 °C, which inhibits pollen development (Mitchell *et al.,* 2016).

The capacity of different plants to adapt to temperature fluctuation underpins their cold and heat tolerance (Ding *et al.*, 2019; De Smet *et al*., 2021). Crucial components of cold stress and heat stress tolerance are the capacities to communicate the ambient temperature change to key tissues, to coordinate metabolic activities to maintain the supply chain for energy production, and to repair or replace proteins and membrane components that are damaged by the stress (Knight and Knight, 2012, Niu and Yang, 2018).

A major factor that reduces vegetative growth of all plant species as temperatures rise is the different temperature response of photosynthesis versus respiration. Net photosynthesis increases as leaf temperature rises, peaking at an optimum temperature and then declining, reflecting the impact of temperature on photosynthetic CO_2_ fixation, and of CO_2_ release by photorespiration and mitochondrial respiration. Wheat leaf respiration increases in response to a short-term rise in temperature (de Vries *et al*., 1979), generally doubling with every 10 °C increase in temperature. The temperature dependence of respiration is probably driven by how temperature affects the processes of substrate supply and demand (Posch *et al.*, 2019). If the increase in temperature is slow, photosynthesis can acclimate so that metabolic rates remain stable, but it if rises suddenly, a heat shock response is initiated.

Photosynthetic and respiratory membranes of plastids and mitochondria differ in composition and in metabolic response to temperature; glycerolipids and phospholipids predominantly occur in the mitochondrial envelope, while galactolipids are predominantly found in the thylakoid membranes of plastids (Yu *et al.,* 2021). This is further complicated by membranes not being a continuous fluid bilayer, but containing membrane microdomains which themselves contain selected protein compositions. The net result of these differences leads to membrane-bound photosynthesis reactions peaking at 30 °C and suffering a slow decline at higher temperatures, while the mitochondrial respiratory rate continue to accelerate to 40–45 °C before a sudden collapse induced by membrane permeability (Posch *et al.*, 2019).

Many substances or protective proteins are synthesized in plants in response to high temperature, including soluble sugars, proline, antioxidants, and protective proteins (Wahid *et al*., 2007; De Smet *et al*., 2021; Zhou *et al.*, 2021). The latter include synthesis of metabolic bypasses and protein-stabilizing chaperones (heat shock proteins) (Waters and Vierling, 2020) that involve both transcriptional programmes and modification of the ribosome for the translation of cold and heat tolerance factors.

A significant and unifying cause of extreme temperature-related imbalances arises from the properties of biological membranes. At low temperatures, but above freezing, biological membranes undergo a phase transition that dramatically alters the capacity of membrane-bound enzyme systems to undertake their function and interact with each other (Fork *et al.*, 1981). At high temperatures, biological membranes undergo a melting process that leads to loss of membrane potential and thus metabolic compartmentation within and between cells (Niu and Xiang, 2018). Plants differ in both the precise temperature of these transitions and the rate of change in their function by the composition of membranes.

Maize being a C_4_ species is sensitive to chilling temperatures (<15 °C), which affects membrane integrity and ROS production. Overexpression of *S*-adenosylmethionine decarboxylase (SAMDC) increased polyamine content and reduced membrane damage. In 3 years of field experiments, the contents of polyamines increased in the leaves of maize plants overexpressing the SAMDC gene, and yield was significantly higher (Jiao *et al.*, 2022).

At very low temperature, cellular acclimation is required to reinitiate growth rate in cold-tolerant plants. Initiation of acclimation involves communication processes associated with cold perception (Ding *et al.*, 2019; De Smet *et al.,* 2021), along with respiratory acclimation to provide ATP synthesis (Atkin and Tjoelker, 2003), re-initiation of ribosomal function for protein synthesis (Beine-Golovchuk *et al.*, 2018), and adoption of cold-tolerant pathways of phloem loading to ensure the supply chain of photosynthates to sinks (Xu *et al*., 2018).

### Flooding stress—soil waterlogging and plant submergence

Roots in a waterlogged soil have no contact with air; this greatly restricts the gas exchange rate, leading to depletion of oxygen supply, accumulation of CO_2_, and lack of dissipation of gaseous hormones such as ethylene. Prolonged waterlogging causes deep roots to die, and laterals to proliferate near the surface. At the same time, intercellular air spaces form in roots. In some species, there is deposition of the hydrophilic polymers lignin and suberin in the walls of subepidermal layers which results in a barrier to radial oxygen loss so that the oxygen that diffuses down the intercellular air spaces from leaves to roots reaches the growing root tips. These changes can be constitutive as in rice and other waterlogging-tolerant species, or induced by waterlogging (Bailey-Serres and Voesenek, 2008).

Severe flooding can cause partial or total submergence of the plant. Inside flooded plants, there is an energy crisis initiated by insufficient ATP production (Gibbs and Greenway, 2003), a carbohydrate crisis owing to lack of supply of photosynthates due to low light and CO_2_ during submergence, and soil toxicities (reduced Mn, Fe, and S). Oxygen-dependent processes such as haem synthesis do not occur during flooding, meaning that new tissues formed during flooding lack important enzyme systems. Damage to cellular machinery mainly occurs upon re-aeration when excessive formation of ROS occurs due to the introduction of oxygen to cells with low energy status and limited antioxidant capacity (Colmer and Voesenek, 2009).

Flooded plants have elevated starch and sucrose catabolism, and glycolysis which supports ethanolic fermentation and produces ATP (Gibbs and Greenway, 2003). Energy production can be further stimulated through alanine metabolism to succinate (Bailey-Serres and Voesenek, 2010). An anaerobic metabolic rate that is too fast leads to depletion of stored reserves and cell death, while a rate that is too slow leads to lack of energy for critical processes. Flooding-tolerant genotypes typically occupy the ‘Goldilocks’ zone where metabolic rate matches stored reserves for a time period typical for flood reversal or for leaves to grow above flood water levels. To do this, plants must reduce energy consumption and growth under low O_2_ conditions to conserve energy (Bailey-Serres and Voesenek, 2010). Anatomical adaptations include the formation of gas films on plant tissues to allow underwater photosynthesis (Pedersen *et al.,* 2009).

A trait within the energy capacity being used successfully by breeders to increase submergence tolerance of rice is the case of the gene *SUB1a*. This transcription factor gene represses GA synthesis and GA-mediated signalling pathways, reducing shoot elongation during flooding, by slowing carbohydrate consumption, preventing chlorophyll breakdown, and activating alternative energy pathways (Xu *et al*., 2006). It slows growth until the flood subsides, conserving energy needed for survival, and has been shown to effectively do this under field conditions. This provides direct benefit to farming communities (Dar *et al.,* 2013; Ismail *et al*., 2013) and opens up new pathways for rice improvement in the face of changing climates (Emerick *et al.,* 2019).

### Nutrient stress—nutrient deficiency and ion toxicity

Nitrogen, phosphorus, and potassium (NPK) are the major nutrients limiting crop growth and yield, while limited availability of micronutrients such as Fe can also be a stress to crops when soil pH is high. Growth rate is reduced to the rate at which the most limiting element can be taken up and assimilated. Roots proliferate in relation to leaves so that the root:shoot ratio increases. Capacities to relieve the stress include the induction of membrane transporters to scavenge ions from the rhizosphere, as well as intracellular signalling, trafficking, and storage inside cells, and the optimization of metabolic processes and recycling between tissues and organs.

Nitrogen in the form of nitrate or ammonium is essential for protein synthesis. Leaves have a particularly high requirement for nitrogen, with up to 30% in the CO_2_-fixing enzyme Rubisco (Evans, 1989). Shoot growth is reduced, and continued root growth results in a higher root:shoot ratio. Multiple nitrate uptake transporters of the NRT1 and NRT2 families work together to enable nitrogen uptake (Schroeder *et al*., 2013). Nitrogen use efficiency was increased in maize by increasing expression of the transcription factor ZMM28, resulting in maize plants with increased plant growth, photosynthesis capacity, nitrogen utilization, and grain yield (Wu *et al*., 2019). Isotope ^15^N labelling demonstrated that transgenic lines had improved nitrogen uptake and nitrogen utilization efficiency which was achieved through both greater nitrogen harvest index and reproductive nitrogen remobilization (Fernandez *et al.*, 2022)

Phosphate is essential for growing tissues for the synthesis of membrane phospholipids and nucleic acids, and in all cells for metabolic processes that involve phosphorylation reactions. Insufficient phosphorus leads to an increased root:shoot ratio. Root growth may be stimulated in comparison with phosphorus-sufficient plants, including proliferation of root hairs which are responsible for taking up most of the phosphorus . A phosphate efflux transporter (PHO1) in the Golgi network is essential for phosphorus transport to the shoot (Schroeder *et al*., 2013). Phosphorus use efficiency was increased in rice through the *PSTOL1* gene. A QTL for tolerance of low soil phosphorus, *PUP1*, was found in rice and was associated with enhanced grain yield on phosphorus-deficient soil (Wissuwa, 2005). Further analysis found the protein kinase gene *PSTOL1* at the *PUP1* locus and showed that its expression enhanced seedling root length and phosphorus uptake in rice (Gamuyao *et al.*, 2012). Homologues in sorghum enhanced phosphorus acquisition and performance on low-phosphorus soils (Hufnagel *et al.*, 2014)

Potassium is the major component of osmotic pressure in cells and is needed for cell expansion in growing tissues and the maintenance of cell volume in mature cells, and for stomatal function. As for nitrogen and phosphorus deficiency, the root:shoot ratio increases, leading to greater ‘exploration’ of soils to maintain a given shoot mass.

Many essential micronutrients are heavy metals normally present in low abundance in soil, although sometimes at high and toxic concentrations in soils contaminated from industrial activity. Zn is one such element, and must be maintained at very low concentrations in the cytosol by organelle membrane transport proteins (Broadley *et al*., 2007).

Aluminium is ubiquitous in soil and becomes toxic to many crop species when soil pH falls below 4.5 because the increased concentration of Al^3+^ cations inhibits root growth. A mechanism of Al^3+^ resistance in many species relies on the release of the organic anions malate and citrate from the root tips which chelate the harmful Al^3+^ (Ryan and Delhaize, 2010). The two main Al^3+^ resistance genes in bread wheat encode transporters TaALMT1 and TaMATE1B which facilitate malate and citrate release, respectively, while the major Al resistance gene in sorghum encodes the transporter SbMATE that releases citrate. When these genes are transformed into Al^3+^-sensitive cereal species by introgression or transgenic techniques, they enhance organic anion release and increase root growth and plant biomass in acid soils (Delhaize *et al.*, 2009; Zhou *et al.*, 2014; Carvalho *et al*., 2015; Kawasaki *et al.*, 2023).

The harmful effects of other toxic metals taken up by roots can be avoided by sequestration in specific cell types (Zhao *et al.*, 2022). Toxicity remains a problem for human consumption of plants accumulating As and Cd in contaminated soils. Transporters have been identified for Cd uptake, vacuolar sequestration, and root–shoot transport, and shown to reduce Cd uptake by the rice grain (Ma *et al.*, 2021).

## Benefits of considering capacities as a framework for stress responses

In this review we have suggested seven capacities that enable plants to adapt to many abiotic stresses. Some key traits arising from these capacities are summarized in Fig. 3. These may help in assessing the relative importance of genes arising from ‘omic’ and QTL studies. Multi-omic network studies and GWAS-defined QTLs provide a large number of genes of interest that if filtered through plant capacity prioritization could speed stacking of traits for glasshouse assessments and field evaluations. Each environmental stress category has a different number and type of capacities (Fig. 3) that may simplify the process of searching for genes underpinning effective tolerance mechanisms.

### Capacities relevant to all stresses

The focus on plant capacities that are common to most, if not all, abiotic stresses can reduce the apparent complexity of the response of different species to different environmental conditions. This is particularly useful as many stresses co-exist in nature, for example drought and high temperature. Figure 3 indicates traits that are common to a number of stresses.

This review has summarized fundamental capacities possessed by all plant species that are critically important in their adaptation to abiotic stresses.

Application of abiotic stress in different species may evoke special examples of responses, and complexity in timed phases of these responses is often reported. At the molecular level, a vast array of responses in transcription, translation, and metabolite accumulation reach into nearly every process in cell and tissue development and maintenance. Depending on the timing of these responses and the plant phenotypes observed, these are variously interpreted as signs of panic, short-term benefits, adaptation, or avoidance. At the physiological level, rapid responses of stomata limit water loss on a temporal basis, whereas short to medium term decreases in cell division rate and cell expansion lead to a slower emergence of new leaves and their reduced final size. These reduce the ability of the plant to grow quickly but limit their water loss. At the anatomical level, changes in cell size and shape, cell developmental patterns, and cell wall composition lead to a reduction in wasteful water loss by leaves and roots.

Sorting out the primary events that drive these responses and lead to acclimation to stress from the secondary, longer term consequences of these events is a difficult task. An example of this is the increase in ROS and antioxidant production which accompanies all stresses, but is likely in many cases to be a secondary response to the stress. As explained earlier, changes in ROS and antioxidants are part of normal plant development and are only significant as a cause of stress if they damage macromolecules. Measurements of peroxidized lipids, oxidized DNA, or precipitated proteins are needed (Box 1).

**Box 1. What is oxidative stress?**

Formerly, oxidative stress on plants was considered an environmental condition akin to UV-B radiation, and industrial pollutants such as ozone and sulfur dioxide.

Currently the term is used to refer to an internal chemical status induced by an abiotic stress which in turn causes an increase in production of reactive oxygen species (ROS). The term implies that it is a metabolic stress, but in fact it is a response to the external abiotic stress which has reduced the metabolic activity and growth rate. Terminology is not so important, but the significance (or not) of changes in ROS and antioxidant levels is important. Frequently an increase in ROS level in a given leaf is taken as a sign that the system is under strain, but increases in ROS are normal as tissues age (Khanna-Chopra, 2012). Likewise, increases in antioxidant activity are normal as tissues age. Both change diurnally and under variable light conditions.

Oxidative stress can be assessed quantitatively as accumulation of unrepaired damage to macromolecules; this is needed as evidence of oxidative damage under stress. This involves the measured accumulation of precipitated proteins, peroxidized lipids, or oxidized DNA. The signature leaf must be chosen carefully, and matched against leaves from control plants at the same stage of development.

In summary, a high ROS production rate or even a high ROS level is not a sign of damage to plants. Actively growing tissues have high levels of both, and they can be a normal sign of metabolic activity. Likewise, elevated levels of antioxidants are not evidence of stress, as those vary continually in different cells and tissues, and in different cell compartments.

### Benefit of capacity analysis

The benefit of intepreting plant adaptations to abiotic stresses in terms of capacities to respond is three-fold. It allows us to focus on expectations and the appropriate experimental conditions, it enables the application of relevant phenotyping and phenomics, and it aids the construction of specific hypotheses that can be tested.

The abiotic stress literature today is provided by scientists imposing a change in a controlled physical or chemical scenario and observing or collecting samples of its short- or long-term consequences. Alternatively, it might involve collecting samples or observations from environments when such events are unfolding naturally. The results of either field or laboratory experiments provide different answers based on the various types of observations and measurements made. During each type of abiotic stress, the response may lead to death, but it might also lead to continued slower and sustained growth and allow the plant to complete its life cycle. Resilience probably requires the accumulative use of plant capacities to adapt, with their additive complementary benefits being the difference, often independent of which is deployed first. If we consider the various capacities of plants to respond to stress, we can perhaps use these insights to understand pathways to resilience or pathways to failure in a more simplified way.

### Cautions

For this process to be optimal, experimental treatments enacted for gene discovery should be as relevant to the abiotic stress experienced as technically possible. The experimental treatment should be realistic and take account of the variable environments in fields in which the crop is grown for human food (Plessis, 2023). Field-grown plants have many morphological and development differences from plants in controlled environments (Poorter *et al*., 2016). Sudden and severe shock treatments are not useful if tolerance mechanisms are sought. Avoiding these extremes ensures that experiments are not just studying membrane damage and metabolic injury and the onset of tissue death. ‘Omics’ approaches to biological analysis offer tools to determine the succession of molecular events taking place in specific tissues and cell types in a plant during the onset of water deficit. However, published information needs to be carefully considered in view of the often drastic protocols used to impose water deficit, such as hyperosmotic stress caused by sudden treatment with mannitol or polyethylene glycol. When moderate and progressive water deficits are applied, such as those compatible with crop production in the field, the changes in transcript abundance or enzyme activities are quite subtle. The same principle applies to flooding, temperature shocks, and salinity stress (Shavrukov, 2013). Plants can acclimate to gradual increases in soil salinity, changes in temperature, and oxygen deprivation (Gibbs and Greenway, 2003), but sudden and extreme changes will kill them. Many helpful articles on experimental design for specific abiotic stresses have been published. For example, guidelines for experimental treatments for many abiotic stresses were detailed by Pooter *et al*. (2012), and the physiological evaluation of impacts of salinity on different traits by Negrão *et al*. (2017).

### Future applications for food security

Gene discovery and its potential application to plant breeding could be accelerated by exploring its function and mechanism in the context of one of these seven capacities. This allows the formation of hypotheses, expectations, and the phenotype to measure to be more accurately defined. This focus provides a context for gene discovery—to place a gene of unknown function in a metabolic pathway or physiological process—which will allow its evaluation for tolerance to an abiotic stress.

‘Omics’ studies, whether transcriptomics, metabolomics, or proteomics, reveal hundreds of genes, metabolites, or proteins that are up-regulated or down-regulated many-fold by stress. Likewise, network studies and GWAS-defined QTLs provide a very large number of genes of interest. The question of which gene to choose for further studies can be helped by placing them into a network or biosynthetic pathway. Finding that this pathway has a crucial role in one of the capacities above would provide confidence that the selected genes that are up-/down-regulated are important in stress tolerance and to consider how different genes could be stacked for added, independent benefit.

The challenge for meeting the increasing global demand for foods for human health is to integrate the appropriate molecular and genetic tools for use by plant breeding companies. The power of innovative agronomic management (e.g. Hunt *et al*., 2018) together with an understanding of fundamental plant capabilities for adaptation to abiotic stresses will enhance the success of this effort.

The presence of GM products in the food supply chain remains a flash point for community concern in many countries. While some countries have approved the commercial production of a limited range of GM crops, the global regulatory landscape remains uncertain about genome-edited plants (Nuccio *et al*., 2018) requiring societal perspectives and public engagement about emerging technologies to be further explored (Bowerman *et al*., 2023). This means that screening natural populations and introgressing traits into food plants by marker-assisted selection still remains the preferred method for cultivar release in many countries at the present time.
